# Preparation and Characterization of pH Sensitive Chitosan/3-Glycidyloxypropyl Trimethoxysilane (GPTMS) Hydrogels by Sol-Gel Method

**DOI:** 10.3390/polym12061326

**Published:** 2020-06-10

**Authors:** Chi-Ping Li, Mao-Chi Weng, Shu-Ling Huang

**Affiliations:** Department of Chemical Engineering, National United University, Miaoli 36063, Taiwan; chipingli@nuu.edu.tw (C.-P.L.); m40817@gmail.com (M.-C.W.)

**Keywords:** chitosan, hydrogels, swelling thermodynamics and kinetics, differential scanning calorimetry (DSC), interaction force parameter

## Abstract

pH responsive chitosan and 3-Glycidyloxypropyl trimethoxysilane (GPTMS) hydrogels were synthesized by the sol-gel crosslinking reaction. GPTMS was introduced to influence several behaviors of the chitosan hydrogels, such as the swelling ratio, mechanical properties, swelling thermodynamics, kinetics, and expansion mechanism. The functional groups of Chitosan/GPTMS hybrid hydrogels were verified by FT-IR spectrometer. Differential scanning calorimetry (DSC) and the thermogravimetric analysis (TGA) were used to analyzed the thermal behavior of water molecules, the expansion of thermodynamics, and the content of water molecules in the hydrogel. The results show that hydrogel consists of 50 wt.% GPTMS (CG50) and has good mechanical properties and sensitivity to pH response characteristics in the acidic/alkaline buffer solution. The increase of GPTMS content leads to the increase of hydrophobic groups in the hydrogel and causes the decrease of the overall water content and the freezing bond water content. When the hydrogels were immersed in acid solution, the interaction force parameter was smaller than that of DI-water and alkaline. It means that the interaction forces between hydrogel and water molecules are relatively strong. The swelling kinetics of hybrid hydrogels were investigated to inspect the swelling mechanism. The result is consistent with the Fisk’s diffusion mechanism, meaning that the rate of water penetration is adjustable. The biodegradable hydrogel (CG50) in this study has good environmental sensitivity and mechanical properties. It is suitable to be applied in the fields of drug release or biomedical technology.

## 1. Introduction

The polymer hydrogel is a polymer material composed of a hydrophilic 3D network structure, which allows the polymer hydrogel to absorb a large amount of solvent and swell, and also has good biocompatibility. The forces that affect the water absorption of hydrogels include hydration, penetration, and capillary phenomena. The swelling balance depends on the relative magnitude of these forces, and to a large extent affects some important properties of hydrogels, including diffusion characteristics, internal transportation, and mechanical properties [1,2,3]. Hydrophilic hydrogels have many unique physical and chemical properties, which make hydrogels widely used in the field of biomedical materials. For example, it is used in the controlled release of drugs. Because of the weak hydrophobic force between the hydrogel and the protein, the macromolecule drug can be effectively coated in the polymer matrix of the hydrogel [4]. Hydrogels are also considered to be biomimetic tissues because they can contain a lot of water inside the structure, and this water can allow small molecules to pass through, promote local and sustained release of drugs, and thus reduce the number of administrations, prevent damage to drugs, and allow relatively low dose delivery. Natural or synthetic polymers can be used to prepare hydrogels [5,6,7]. Natural polymer hydrogels can provide sufficient biocompatibility, biometrics, biodegradability, and other functions, but they cannot provide sufficient mechanical strength. Synthetic polymer hydrogels do not have fixed biological activity characteristics, but the mechanical properties of synthetic polymer hydrogels are better than natural polymer hydrogels. There are many classification methods for hydrogels, mainly depending on their physical properties, swelling properties, polymerization composition, preparation method, ionic charge, crosslinking method, and biodegradability [8]. Hydrogel materials will change their characteristics due to environmental stimuli. When hydrogels are physically or chemically stimulated, they will swell or shrink (also known as intelligent hydrogels). These environmental factors include temperature, pressure, pH value, magnetic field, electric field, optical radiation, and ultrasonic radiation [9,10]. The stimulus-responsive hydrogel will give different feedbacks due to changes in the external environment, such as oxygen permeability response, volume change, mechanical strength, etc. Physical stimuli include temperature, light source, pressure, electric and magnetic fields, etc., changing the intermolecular interactions that occur at the critical point. The chemical stimulus contains the pH value, chemical agents, and ions, and it responds to the interaction between the functional groups contained in the polymer chain and the solvent. The different response behaviors of hydrogels can be divided into the several types [11], like temperature-sensitive hydrogel [12,13,14,15,16,17,18], pH-sensitive hydrogel [6,19,20,21,22,23,24], ionic strength hydrogel [19], glucose sensing hydrogel [25], and light-sensitive hydrogel [26]. A pH-sensitive hydrogel is a polymer hydrogel that can change in volume with changes in the environmental pH and ionic strength. The interaction forces that cause the volume change of the pH-sensitive hydrogel are mainly the forces between ions and hydrogen bonds. Its molecular structure usually has an ionizable acid (carboxy) group (–COOH or –SO_3_H) or an amino group. With the change of environmental pH, the group will dissociate, making the hydrogen between the polymer chains in the structure. The bond dissociates, and the force between the ions increases the electrostatic repulsion, resulting in swelling and de-swelling behavior. Generally, pH-sensitive hydrogels can be divided into three different response types, which are anionic, cationic, and zwitterionic [13,27]. The sensitive group of the anionic pH-sensitive hydrogel is generally –COO^–^. This type of hydrogel is generally in a contracted state in a low-pH environment, because in a low-pH environment, the dissociation degree of the ionizable groups of the hydrogel is low, making the electrostatic repulsion force on the gel swelling not contribute. When the pH value of the environment increases, the electrostatic repulsion between the carboxylate groups increases, and the electrostatic repulsion causes the expansion rate of the hydrogel to increase. The sensitive groups of cationic pH-sensitive hydrogels are generally basic amine groups, and their pH sensitivity mainly comes from the protonation of amino groups. The more amino groups, the stronger the hydration of the hydrogel, the greater the electrostatic repulsion between the protonated amino groups, resulting in a greater degree of equilibrium swelling [20]. Zwitterionic pH-sensitive hydrogels also have acid–base groups. Its pH sensitivity comes from the ionization of two groups in the hydrogel network. Basic groups will ionize in low-pH environments. The acidic group is ionized in a high-pH environment, so the zwitterionic hydrogel has a relatively large electrostatic repulsion in high- or low-pH environments, so it has a large swelling capacity and has relatively smaller swelling ability in a moderate environment. Therefore, this type of hydrogel has good swelling ability in almost all pH ranges, and it is more sensitive to the changes of ionic strength.

The swelling step of the hydrogel can be divided into two stages. The first step is that the solvent molecules enter the hydrogel and interact with the network polymer chains to form a hydrated layer. Then, the second stage is that the solvent molecules continue to penetrate. At this time, the volume of the hydrogel will increase greatly, and the weight of the hydrogel at this time can reach dozens of times the weight of the dried hydrogel. Because of the dissociation effect of ionic groups (such as –NH_2_, –COOH, and –SO_3_H) in the structure, the ionic hydrogel increases the hydrophilicity of the hydrogel, resulting in its strong water absorption capacity. At the same time, the increase in the degree of dissociation further extends the polymer chains in the network and makes sufficient contact with water molecules. The properties of the polymer itself (hydrophilicity/hydrophobicity and crosslinking degree) and the degree of dissociation of hydrogel functional groups and various factors of ionic hydration (environmental factors such as temperature, pH, ionic strength, ionic properties, and swelling medium composition) will affect the swelling ability of the hydrogel.

The water molecules in hydrogel polymers can be divided into three different forms: (1) Free water, also known as intermediate water or free water, is usually distributed on the surface of the polymer and is easily lost. Its thermodynamic behaviors, such as crystallization, melting temperature, and enthalpy change, are similar to those of pure water. (2) Non-freezing bond water, which is water that is bonded to the polar hydrophilic group of the polymer network structure through hydrogen bonding, has no phase change within the temperature range of −100~−50 °C, and its crystallization exothermic or melting endothermic is usually not observed; it is more closely bonded to the polymer matrix. (3) Freezing bond water’s phase transition temperature is usually lower than 0 °C; the water in this state in the hydrogel should be weakly bond water. Non-freezing bond water surrounds the water in the bond water network with a certain orientation, forming a second or third hydration layer. Although the bonding effect of the freezing bond water is slightly weaker than that of the non-freezing bond water, it is not easy to remove [28,29,30,31,32].

The bulk water content is the sum of the content of freezing bond water and non-freezing bond water. Bonded water has a great influence on the structure and function of hydrogels and also plays an important role in the process of biological metabolism. Many scholars use Differential Scanning Calorimetry (DSC) to detect the presence of water in different polymers [28]. By analyzing the melting endothermic peaks of polymer that can be frozen near 0 °C, the ratio of the enthalpy value (ΔH_fm_) to the endothermic peak enthalpy value (ΔH_0_) of pure water at 0 °C can be used to calculate the polymer’s freezing water content (W_fm_). The bulk water content (BWC) of the polymer is the sum of freezing water content (W_fm_) and non-freezing water (W_nf_), so the amount of water in different states in the polymer can be obtained, as expressed by the following formulas:

(1) $$W_{\mathrm{fm}}(\%)=\frac{\Delta H_{\mathrm{fm}}}{\Delta H_{0}}\times 100$$

(2) $$\mathrm{BWC}(\%)=\frac{W_{\mathrm{wet}}-W_{\mathrm{dry}}}{W_{\mathrm{wet}}}\times 100$$

(3) $$W_{\mathrm{nf}}(\%)=\mathrm{BWC}-W_{\mathrm{fm}}$$

where W_wet_ is the overall water content of the hydrogel when it reaches equilibrium, and W_dry_ is the weight of the hydrogel polymer when it is dry.

When water is in contact with the hydrogel network structure, the hydrogel polymer chain will interact with the solvent molecules, causing the hydrogel network structure to swell, and this swelling behavior can be calculated by using the Flory–Rehner theory [33] thermodynamics of the mixing process. It can be proved by combining the theory of rubber elasticity and thermodynamics that the equilibrium swelling is mainly controlled by two opposing forces, which are (1) the mixing force between the hydrogel and the solvent, and (2) the high hydrogel of the contraction force of the molecular chain; when these two opposing forces reach equilibrium, the two forces can cancel each other out [34,35]

The interaction constant χ value (interaction force parameter) can be regarded as the interaction index between the polymer gel and the solvent, and it can be roughly regarded as the hydrophilicity and hydrophobicity of the polymer in the solvent. The force parameters are derived from the Flory–Huggins theory [36]. After subsequent research, the force parameter expression can be obtained from Equation (4), which can be expressed by the effective swelling balance.

(4) $$\phi_{2}+\ln(1-\phi_{2})+\chi \phi_{2}{}^{2}+\upsilon_{e}V_{1}(\phi_{2}{}^{\frac{1}{3}}-2\phi_{2}f^{-1})=0$$

where *ϕ*_2_ is the volume fraction of the polymer when it expands, *V*_1_ is the molar volume of the solvent, *υ_e_* is the effective crosslink density, and *ƒ*^−1^ is the average number of active elastic chains. The magnitude of the force parameter is related to the contribution of the mixed chemical potential energy, so from a numerical perspective, the elastic contribution only has a slight effect on the force parameter, because the value of *υ_e_* in the system is very small, and it can even be ignored, in order to obtain Equation (5).

(5) $$\phi_{2}+\ln(1-\phi_{2})+\chi \phi_{2}{}^{2}\approx 0$$

Finally, the force parameter between the polymer and the water molecule can be calculated by Equation (6).

(6) $$\chi =\frac{1}{2}+\frac{\phi_{2}}{3}$$

The behavior of water molecules diffusing into the hydrogel network and the relaxation of the polymer chains can be used to explain the dynamic behavior of the gel during swelling [37,38].

Fick’s model is suitable for lamellar hydrogel films. When the water content of the hydrogel film is not large, and the macromolecular chain relaxation rate between the network-like structures is fast, the swelling process of the hydrogel film is mainly controlled by the water molecule diffusion process, which can be obtained by using Fick’s diffusion equation description:

(7) $$\frac{M_{t}}{M_{\infty }}=kt^{n}$$

where *M_t_* is the weight measured under a fixed time interval, *M_∞_* is the weight measured when swelling reaches equilibrium, *k* (characteristic constant) is a constant describing the structure or geometry of the hydrogel, *t* is time, and *n* (mechanism exponent) indicates the transport mode of the solvent into the gel. By plotting the slope and intercept of log (*M_t_*/*M_∞_*) vs. log (*t*), we obtain the values of *n* and *k*, respectively.

According to the calculated value of *n*, it can be divided into three different transmission behaviors:

(1) When the calculated value of *n* is less than or equal to 0.5 (*n* ≤ 0.5), it is called Fick’s diffusion transmission behavior. In this case, the diffusion rate is much greater than the relaxation rate of the hydrogel polymer chain. In other words, the interaction between the molecules of the hydrogel network structure and the solvent is much larger than the interaction between the molecular chains of the gel network structure, so that the polymer chain of the hydrogel can quickly relax and expand. Thus, this system is a diffusion control process.
(2) When the calculated value of *n* = 1.0 (*n* = 1.0), it is an extreme transmission behavior. In this case, the relaxation rate of the hydrogel polymer chain is much greater than the diffusion rate, meaning that the interaction between the hydrogel network molecular chain is much greater than the interaction between the hydrogel network molecular chain and the solvent. Therefore, the system is controlled by the speed of hydrogel swelling.
(3) When the calculated value of *n* is in the range of 0.5~1.0 (*n* = 0.5~1.0), it is called non-Fick’s transmission behavior. It means that, during the swelling process of the hydrogel, the relaxation rate of the polymer chain is equivalent to the diffusion rate of the solvent.

The structure of chitosan has a primary amine group and a first- and second-order hydroxyl group, and its pka is about 6.5. This property allows it to have different swelling and de-swelling behavior in an acid or alkali environment. In other words, chitosan is also one of the pH-sensitive materials. However, although chitosan has good biocompatibility and degradability, when it is used as a drug-release material, it is too sensitive to pH and it is difficult to control. Therefore, it can be physically blended or connected by using a crosslinking agent or a polymer. To enhance mechanical properties to improve shortcomings and increase drug-release time, Chitosan itself contains a hydrophilic hydroxyl group (–OH) and a more reactive nucleophilic amine group (–NH), which is prone to high water swelling, and in an acidic environment, the structure is easily damaged, so a crosslinking agent is often used for crosslinking reaction to increase its mechanical properties and acid resistance [39,40]. However, chitosan can provide more application options after physical or chemical crosslinking [41,42]. Because chitosan is positively charged, has active functional groups, and has excellent biodegradability and biocompatibility, research on chitosan can be seen in disease treatment and drug release [43,44,45,46]. There are many other applications, such as external dressing [47], protein separation [48], biosensor [49], tissue engineering [50], and biochips [51,52,53,54,55,56,57,58].

**Figure 1:**
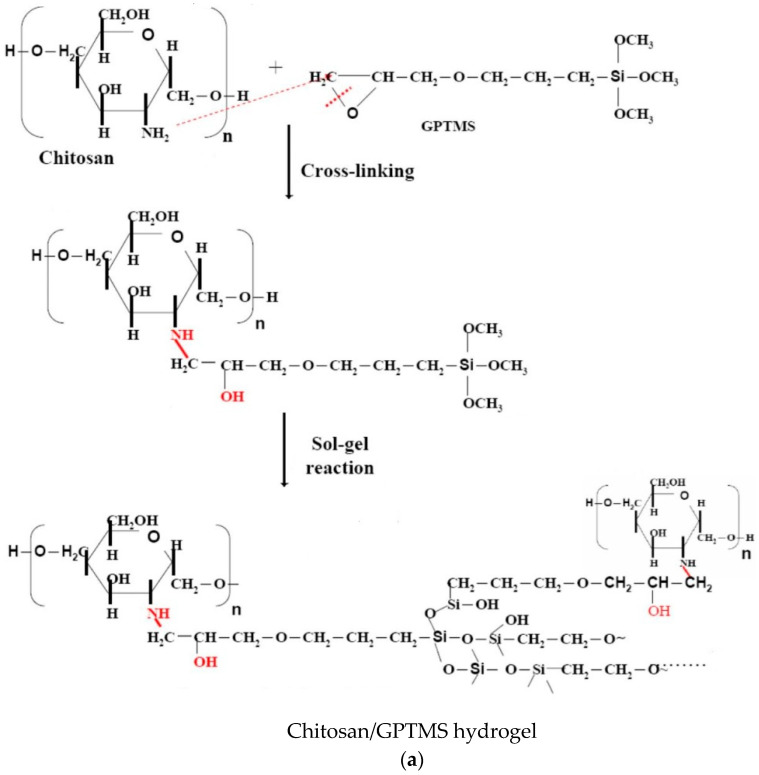

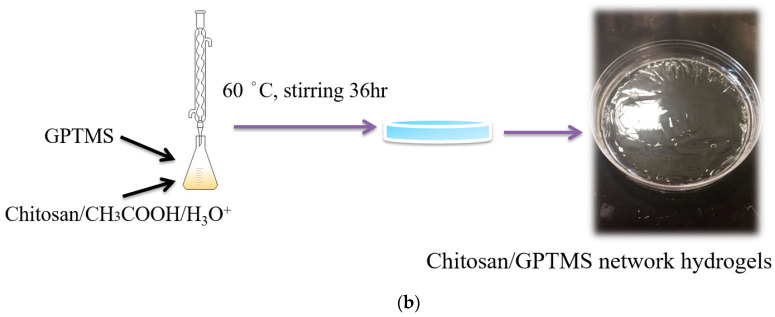
(**a**) Synthesis reaction of Chitosan/GPTMS hydrogel. (**b**) Preparation of Chitosan/GPTMS hydrogel film.

**Figure 2:**
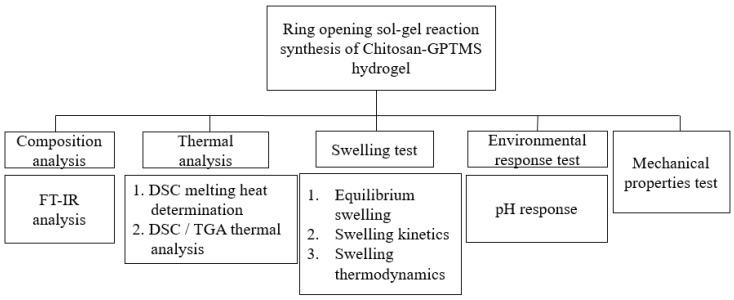
Research method summary.

The design of this study mainly uses GPTMS as the crosslinking agent to carry out ring-opening and sol-gel crosslinking reaction with chitosan (Figure 1). It is hoped that the crosslinking reaction can increase the mechanical properties and acid resistance of chitosan hydrogel. Various characteristic analyses such as water swelling ratio, DSC thermal analysis, pH response test, expansion heat, and kinetics, to analyze the characteristics of different proportions of GPTMS additions, were carried out (Figure 2). The result shows that Chitosan/GPTMS hybrid hydrogel is suitable for pH-sensitive hydrocolloid materials.

## 2. Materials and Methods

### 2.1. Materials

The chitosan, 3-Glycidoxypropyltrimethoxysilane (GPTMS, Mw = 236.34 g/mol), acetic acid (purity 99.7%), sodium hydroxide (purity 95%), and hydrochloric acid (purity 37.4%) were purchased from Sigma-Aldrich (Taipei, Taiwan), Acros (Toufen, Miaoli), Showa Company Shimakyu’s pure chemical Company (Osaka, Japan), and Fisher Chemical (Pittsburgh, PA, USA), respectively.

### 2.2. Preparation of Hydrogel Thin Film

We prepared 5 bottles of chitosan solution through dissolving one gram of chitosan in 5% acetic acid solution in each bottle and stirring at room temperature for 24 h. GPTMS was then added into the chitosan solution to form 10%, 25%, 50%, 75%, and 90% of GPTMS (wt.%) solution, and we stirred at 60 °C for 36 h, respectively [59]. The reacted solution was poured into a PP plastic petri dish and dried at 40 °C for 48 h. We remove the synthesized hydrogel from the Petri dish, soaked the film in 0.1 M NaOH for 10 min, to remove acid radicals, and stored it in ultra DI water one day, for testing. The structure of resulted chitosan/GPTMS films was investigated by FT-IR (Jasco FT/IR-470, Tokyo, Japan).

### 2.3. Characterization

#### 2.3.1. Hydrogel Equilibrium Swelling Experiment

First, we wiped off the water on the surface of the hydrogel film that had reached the swelling balance, and then we performed the first weighing. The weight obtained was the wet weight of the hydrogel (W_wet_). Secondly, we dried the saturated hydrogel film in an oven at 105 °C for 12 h. Thirdly, we removed the dried hydrogel film, weighed it, and recorded the dry weight (W_dry_) of the hydrogel.

By substituting the measured wet weight (W_wet_) of the hydrogel and the dry weight (W_dry_) of the hydrogel into Equation (8), we obtain the equilibrium swelling ratio.

(8) $$\mathrm{Swelling}\;\mathrm{ratio}=\frac{W_{\mathrm{wet}}{-W}_{\mathrm{dry}}}{W_{\mathrm{dry}}}$$

#### 2.3.2. Hydrogel Environmental Response Test

In the pH response test experiment, the dried Chitosan/GPTMS hydrogel film was alternately placed in a hydrochloric acid/sodium hydroxide solution of pH 4 and pH 12, and after about 30 min, it was taken out and wiped to dry the surface, and weighed. The films were placed in the acidic and basic buffer solution for 90 min, and after being repeated several times, it was substituted into Equation (8), to obtain its swelling ratio.

#### 2.3.3. Analysis of Thermal Behavior of Water in Hydrogel by DSC

We weighed about 5 mg of the hydrogel sample that reached equilibrium and swelling, wiped off the surface moisture with a filter paper, placed the sample in an aluminum pan, sealed it, and placed it in DSC (TA DSC Q10, Taipei, Taiwan). First, the sample was equilibrated at −25 °C for 30 min, then heated to 25 °C at a rate of 3 °C/min, with the nitrogen flow rate to 30 mL/min, and a DSC spectrum was obtained.

Then, assuming that the enthalpy change of water in the hydrogel is equal to ΔH_0_, the enthalpy of pure water, we substituted the enthalpy (ΔH_fm_) of the endothermic peak near 0 °C in the DSC spectrum into Equation (1). The amount of freezing water (W_fm_), which is the content of bonded freezing water and freezing water, can be found.

The bulk water content (BWC) can be calculated by Equation (2). After deducting the freezing bond water content (W_fm_) from the bulk water content (BWC), non-freezing bond water (W_nf_) in the hydrogel film can be obtained, as shown in Equation (3).

#### 2.3.4. Analysis of Hydrogels by DSC/TGA

We weighed a sample of about 10 mg of hydrogel with equilibrium and swelling, wiped off the surface moisture with a filter paper, placed the sample in a crucible, and placed it in DSC/TGA (TA Q600 SDT, Taipei, Taiwan). After equilibrating at 105 °C for 60 min, we lowered the temperature to 25 °C for 30 min, heated to 700 °C with a heating rate of 5 °C/min, set the nitrogen flow rate to 30 mL/min, and then obtained a DSC/TGA thermal analysis spectrum.

### 2.4. Swelling Structure Parameters of Chitosan/GPTMS Hydrogel

The nano-network structure of crosslinked hydrogels can generally be described by four parameters: (1) volume fraction of swelling polymer (*ϕ*_2_), (2) molecular weight between crosslinking points (M_c_), (3) mesh size (ξ), and (4) porosity (ε) [59,60]. The polymer volume fraction of the swelling hydrogel is described in the amount of liquid contained in the hydrogel. The measured weight of the equilibrium swelling and the weight of the dried hydrogel can be brought into Equation (9), in order to calculate the swelling polymer volume fraction (*ϕ*_2_)

(9) $$\phi_{2}=1-\frac{(W_{wet}-W_{dry})/\rho_{solution}}{W_{dry}/\rho_{polymer}+(W_{wet}-W_{dry})/\rho_{solution}}$$

where *W_wet_* is the mass (g) of the hydrogel when it absorbs water to reach equilibrium; *W_dry_* is the mass (g) of the hydrogel when it is dry; *ρ_solution_* is the density of the solvent; and *ρ_polymer_* is the density of the polymer.

The molecular weight (M_c_) and mesh size (*ξ*) between the crosslinking points in the hydrogel network structure are estimated from the equations proposed by the Flory–Rehner equilibrium swelling theory [60]:

(10) $$\frac{1}{M_{c}}=\frac{2}{M_{n}}-\frac{\frac{\bar{\nu }}{V_{1}}\times [\ln(1-\phi_{2})+\phi_{2}+\chi \phi_{2}{}^{2}]}{\phi_{2}{}^{\frac{1}{3}}-\frac{\phi_{2}}{2}}$$

Among them, M_n_ is the number average molecular weight of the linear polymer chain (Chitosan = 342,500 g/mol), $\bar{v}$ is the polymer specific volume (Chitosan = 0.57 cm^3^/g), and V_1_ is the mole volume of the solvent (H_2_O = 18 cm^3^/mol).

(11) $$\xi =\ell \times \phi_{2}{}^{-\frac{1}{3}}\times {\left(C_{n}\frac{2M_{c}}{M_{r}}\right)}^{\frac{1}{2}}$$

where *l* is the carbon–carbon chain length (1.54 Å), C_n_ is the Flory characteristic ratio (Chitosan = 19), and M_r_ is the repeating unit molecular weight (Chitosan = 161 gmol) [61].

The calculation of the porosity of the hydrogel is as follows:

(12) $$\varepsilon =\frac{W_{\mathrm{wet}}-W_{\mathrm{dry}}}{W_{\mathrm{wet}}}\times \frac{\rho_{\mathrm{sp}}}{\rho_{\mathrm{solution}}}$$

where W_wet_ is the weight (g) when the hydrogel reaches the swelling equilibrium, W_dry_ is the weight (g) of the dried hydrogel, *ρ*_sp_ is the polymer density at the time of swelling (Chitosan = 1.63 g/cm^3^), and *ρ*_solution_ is the solvent density (H_2_O = 0.9963 g/cm^3^).

### 2.5. Calculation of Interaction Force between Chitosan/GPTMS Hydrogel and Water Molecules

According to Flory–Huggins theory and subsequent data projections, the interaction force parameter (χ) between the water molecule and the polymer can be expressed by the effective swelling equilibrium equation, as shown in Equation (4).

The weight of the dried hydrogel and the weight of the hydrogel in equilibrium swelling can be taken into Equation (13), to obtain *ϕ*_2_.

(13) $$\phi_{2}=1-\frac{(W_{wet}-W_{dry})/\rho_{solution}}{W_{dry}/\rho_{polymer}+(W_{wet}-W_{dry})/\rho_{solution}}$$

where *W_wet_* is the mass (g) of the hydrogel when it absorbs water to reach equilibrium; *W_dry_* is the mass (g) of the hydrogel when it is dry; *ρ_solution_* is the density of the solvent; and *ρ_polymer_* is the polymer density.

The values of the force parameter and chemical potential energy contribution of water molecules and polymers can be examined that the elastic contribution only slightly affects the force parameter because the *υ_e_* in the system is very small, as shown in Equation (5). Therefore, the force parameter equation can be simplified, as shown in Equation (6).

### 2.6. Chitosan/GPTMS Hydrogel Swelling Kinetics Test

After drying the hydrogel film in an oven, we recorded the dry weight of the hydrogel film, then immersed the films of different formulas in ultrapure water at room temperature, and then removed the hydrogel every 5 min. We wiped off the surface moisture with a filter paper, and the wet weight of the hydrogel was obtained. In this way, the relationship between the equilibrium swelling rate of the hydrogel and the hydrogel of each formula could be obtained by repeating the experiment until the water content of hydrogel reached saturation. Next, in order to better understand the dynamic mechanism of solvents and hydrogels, Equation (7) was used to fit the initial (i.e., *M_t_*/*M_∞_* ≤ 0.6) swelling behavior of hydrogels.

### 2.7. Chitosan/GPTMS Hydrogel Mechanical Properties Test

Increasing the mechanical properties of Chitosan/GPTMS hydrogels is one of the main points of this study. Because Chitosan hydrogels have poor acid resistance and abilities to protect themself against environmental changes, the addition of GPTMS increases the degree of crosslinking, to improve its mechanical properties.

First, the tensile area and thickness of the hydrogel were measured, and then we used a tensile strength tester (AI-7000 S, Gotech testing machines Inc., Taichung, Taiwan) to measure the mechanical strength of the hydrogel, at a test speed of 1 cm/min. The obtained elastic coefficient and tensile strength could be used to determine the mechanical strength of the hydrogel. The larger the two values, the better the mechanical properties of the hydrogel.

## 3. Results and Discussion

### 3.1. Synthesis and Characterization of pH-Sensitive Chitosan/GPTMS Hydrogels

In this study, GPTMS was used as a crosslinking agent, to perform a crosslinking reaction (Figure 1a) with chitosan, which has poor acid resistance to form a pH-sensitive hydrogel with good mechanical properties, using the method in Figure 1b. The main purpose here is to identify the structure of the synthesized Chitosan/GPTMS hydrogel film and discuss the film characteristics. First, the structure of the synthesized Chitosan/GPTMS hydrogel film was identified by FT-IR spectrometer, the crosslinking situation and the existence of characteristic functional groups were confirmed, and then the degree of swelling of the Chitosan/GPTMS hydrogel film was tested. The DSC and TGA were used to determine the thermal behavior state of water molecules, inorganic portion, and thermal decomposition temperature (T_d_) in the Chitosan/GPTMS hydrogel film. The swelling behavior of the hydrogel under different pH conditions was analyzed, in order to observe the effects of different degrees of hydrogel crosslinking, which cause different network structures on the swelling behavior of hydrogel films.

#### 3.1.1. FT-IR Structure Analysis of Chitosan/GPTMS Hydrogel

**Figure 3:**
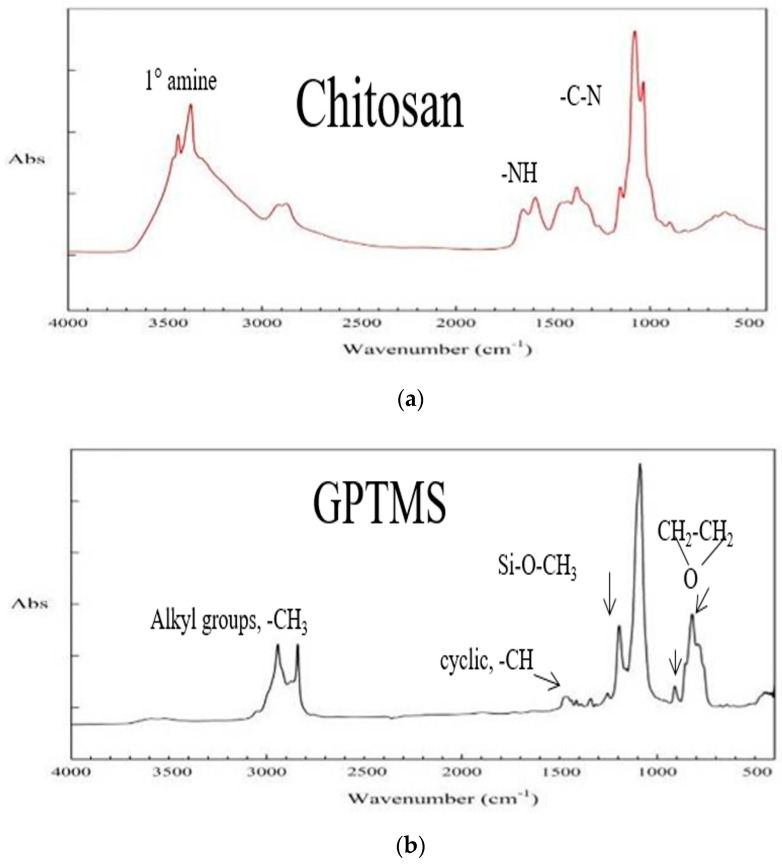

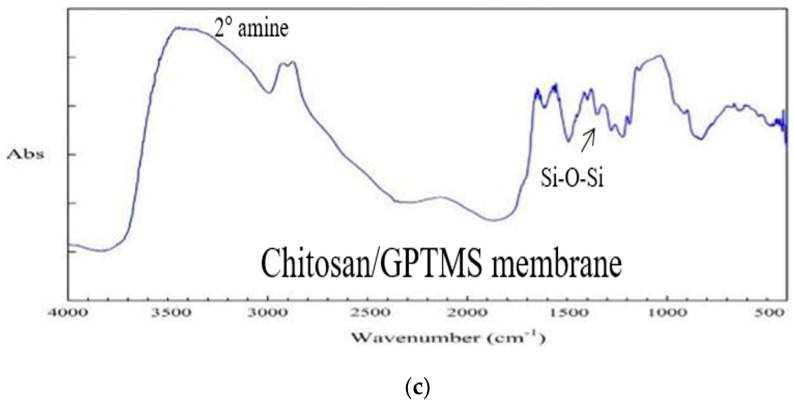
Infrared spectrum of a synthetic hydrogel film and its monomers (**a**) Chitosan; (**b**) GPTMS; (**c**) Chitosan/GPTMS hydrogel.

The Chitosan/GPTMS hydrogel and pure Chitosan and pure GPTMS were scanned by an FT-IR spectrometer, at a range of wavenumber from 4000 to 400 cm^−1^, and the absorption peaks of their functional groups were measured to determine whether the crosslinking was successful. The results are shown in Figure 3.

Pure chitosan films have broad absorption bands, from 3200 to 3500 cm^−1^, which are mainly the stretching vibrational absorption peaks of the primary amine (–NH_2_) and hydroxyl (–OH) groups in the chitosan structure, which is located at 3433 and 3364 cm^−1^ and has two adjacent shoulder peaks, which are characteristic absorption peaks of typical primary amine groups. The stretching vibration characteristic absorption peaks of alkyl (–CH_2_) are around 2882 cm^−1^. The peaks of amine groups bending vibration characteristics of the chitosan structure are at 1662 and 1567 cm^−1^, as shown in Figure 3a.

The methylene group (–CH_2_) of the pure GPTMS epoxy group has characteristic absorption peaks at 905 and 810 cm^−1^, and two strong absorption peak signals at 1183 and 1066 cm^−1^. These are the characteristic absorption peaks of siloxyalkyl group (Si–O–CH_3_) in GPTMS. The peaks at 2950 and 2835 cm^−1^ are the stretching characteristic absorption peaks of alkyl–CH, –CH_2_, and –CH_3_, as shown in Figure 3b.

Figure 3c is the Chitosan/GPTMS hydrogel IR spectrum after the crosslinking reaction. The IR spectrum is similar to pure chitosan. The main difference is that, after the crosslinking reaction, Chitosan’s primary amine group (C–NH_2_) performed a ring-opening reaction with GPTMS to form a secondary amine group (C–NH–C), and only a wide single peak appears at 3433 and 3364 cm^−1^. In addition, the absorption peak of the methylene group of the GPTMS epoxy structure also completely disappeared, proving that the ring-opening crosslinking reaction was successful. After the sol-gel reaction, the characteristic absorption peak of the siloxyalkyl group (Si–O–CH_3_) of GPTMS also disappeared, and the peak was replaced by characteristic absorption of the (Si–O–Si) bond at 1092 cm^−1^.

After FT-IR spectrum analysis, it can be confirmed that Chitosan/GPTMS crosslinked hydrogel can be successfully synthesized by the ring-opening reaction and sol-gel reaction of chitosan and GPTMS.

#### 3.1.2. Equilibrium Swelling Capacity of Chitosan/GPTMS Hydrogel Film

The hydrogels (CG10, CG25, CG50, CG75, and CG90) made in different weight percent of GPTMS were dried first and then placed in DI water, at room temperature, for one day, and measured the weight before and after swelling, to obtain the degree of swelling of hydrogel. From the experimental results (Table 1), it can be found that, when the added GPTMS is 10 wt.%, compared with other hydrogels with lower GPTMS content, it can exhibit a better degree of swelling. The reason is that the Chitosan hydrogel itself has a good swelling ability because it contains hydrophilic hydroxyl (–OH) and amine groups. As more content of GPTMS is added to crosslink with Chitosan, the degree of crosslinking of the hydrogel will increase, and Chitosan will form a Si–O–Si bond with [–Si(OCH_3_)_3_] at the other end of GPTMS. As a result, the hydrophobic groups between the hydrogel networks are increased, which further reduces the hydrophilicity of the Chitosan/GPTMS membrane, thereby suppressing the swelling capacity of the hydrogel film.

#### 3.1.3. DSC Thermal Analysis Results of Chitosan/GPTMS Hydrogel Films with Different Weight Percent of GPTMS

**Figure 4:**
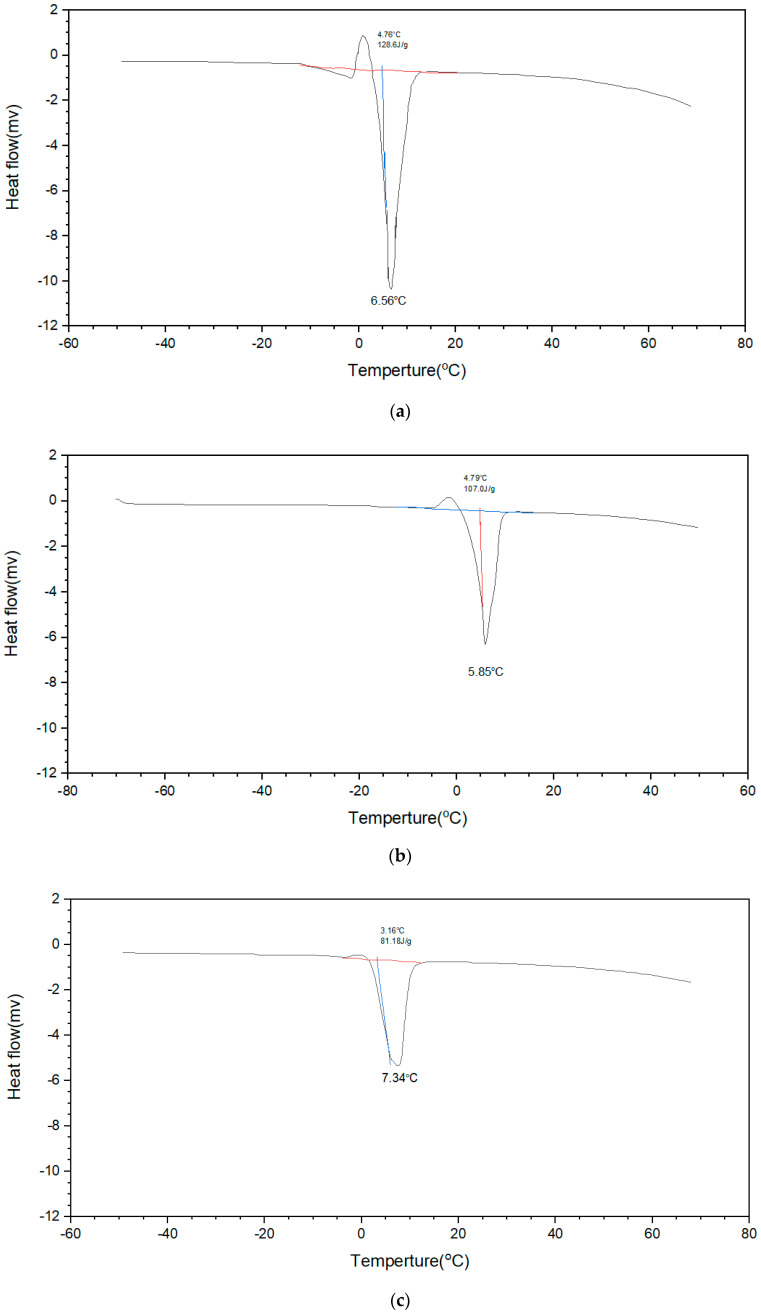

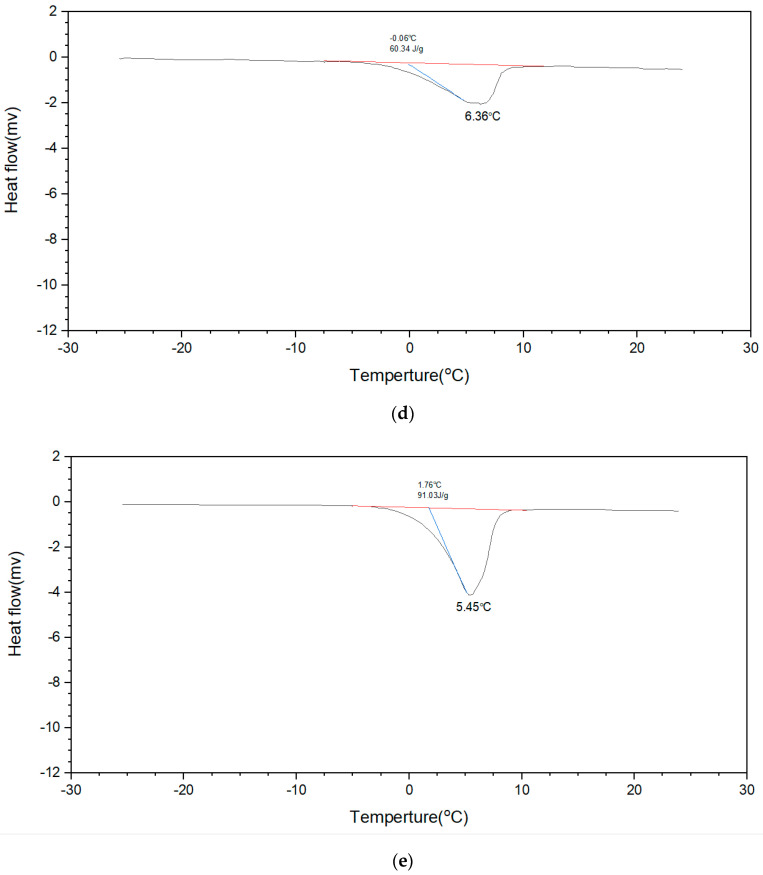
DSC heating curves of Chitosan/GPTMS hydrogels with different proportions: (**a**) CG10; (**b**) CG25; (**c**) CG50; (**d**) CG75; (**e**) CG90.

Figure 4 shows the DSC heating curve of hydrogel films with different ratios of GPTMS. The temperature was heated from −25 to 25 °C, at a rate of 3 °C/min, and Chitosan/GPTMS hydrogel film water absorption capacity and state when it reached the swelling equilibrium were judged by the magnitude of the heat absorption peak near melting 0 °C. From the experimental results (Figure 4), it can be found that the higher the proportion of GPTMS, the smaller the apparent endothermic peak of melting heat, indicating a lower degree of water absorption and swelling. The main reason is speculated that, as more GPTMS is added to crosslink with Chitosan, the degree of crosslinking of the hydrogel will increase, and Chitosan and GPTMS will cause the increase of hydrophobic groups of inter-hydrogel networks due to sol-gel reaction. Therefore, the hydrophilicity of Chitosan/GPTMS film is reduced and thus suppresses the swelling capacity of the hydrogel film.

#### 3.1.4. DSC/TGA Thermal Analysis Results of Chitosan/GPTMS Hydrogels

**Figure 5:**
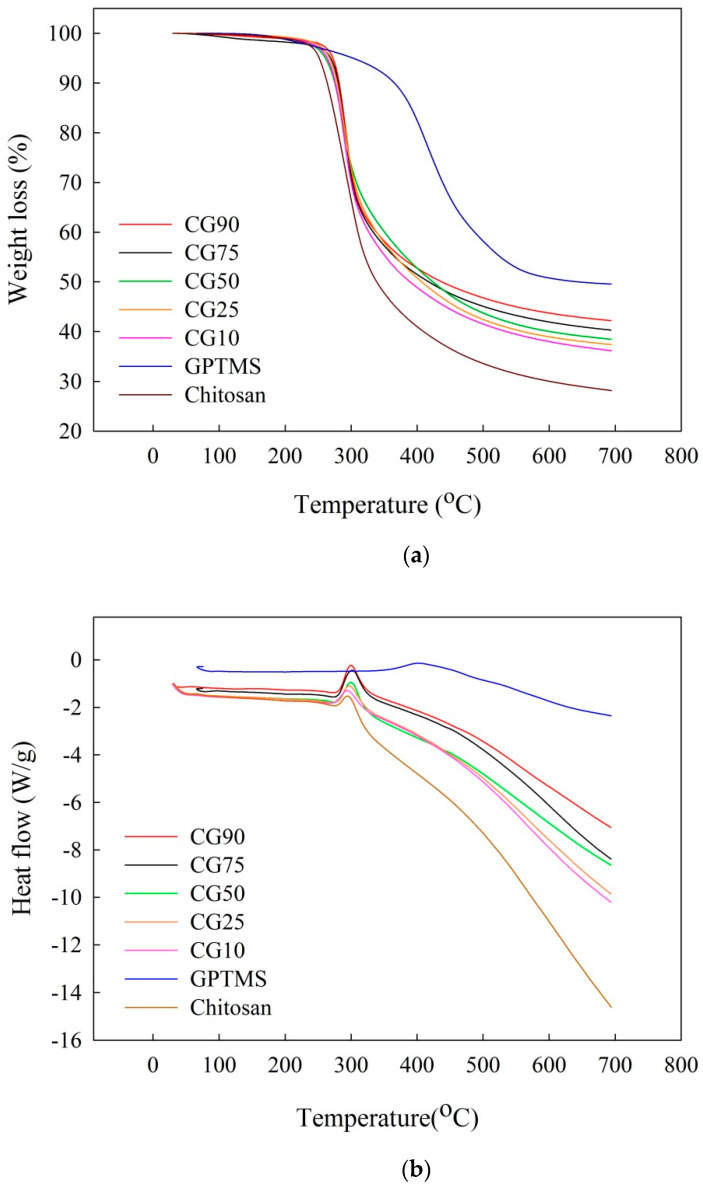
Thermal analysis of hydrogels with different proportions: (**a**) TGA and (**b**) DSC.

Figure 5 shows the DSC/TGA heating curves of different GPTMS weight percent of hydrogels under a nitrogen environment. Through the TGA spectrum, the cracking temperature (temperature at 5% weight loss) of the pure Chitosan film and Chitosan/GPTMS mixed film can be found. This is the film caused by thermal cracking of the organic portion. After the organic phase is cracked, the remaining is the inorganic portion. It can be found that the film made by adding more GPTMS will leave a higher proportion of inorganic phase after firing, because more GPTMS is added, and a result of the increased SiO_2_ content is the hydrogel. In addition, the thermal decomposition temperature (T_d_) of the mixed hydrogel is also relatively with GPTMS amount increased, as shown in Table 2.

#### 3.1.5. Effect of pH on Chitosan/GPTMS Hydrogel

In the Chitosan/GPTMS hydrogel, the chitosan polymer chain is entangled in the silicone network, and the dipole–dipole force is connected, so the water permeability increases with the hydration force of the sample and in response to different pH environments.

The hydrogels (CG10, CG25, CG50, CG75, and CG90) made in different proportions were first dried and dehydrated, and then placed in a solution of pH = 4 and pH = 12, respectively, and equilibrated at room temperature, for one day. The weight before and after swelling was measured, to obtain the swelling degree of the hydrogel. From the experimental results (Table 3), it can be found that the hydrogel has a good equilibrium swelling capacity when placed in an acidic environment. It is speculated that the external H^+^ ions enter the hydrogel and combine with –NH and form –NH_2_^+^ under low pH. As a result, the repulsive force in the polymer network is increased and causes the molecular chain to be in a stretched state. Due to the extension of the polymer chain, the swelling property of the hydrogel is increased, and it can produce a significant swelling phenomenon. Conversely, when the hydrogel is placed in an alkaline environment, the deprotonation phenomenon occurs inside the hydrogel, causing the polymer chain to shrink, and thus causing the hydrogel to exhibit a de-swelling shape.

**Figure 6:**
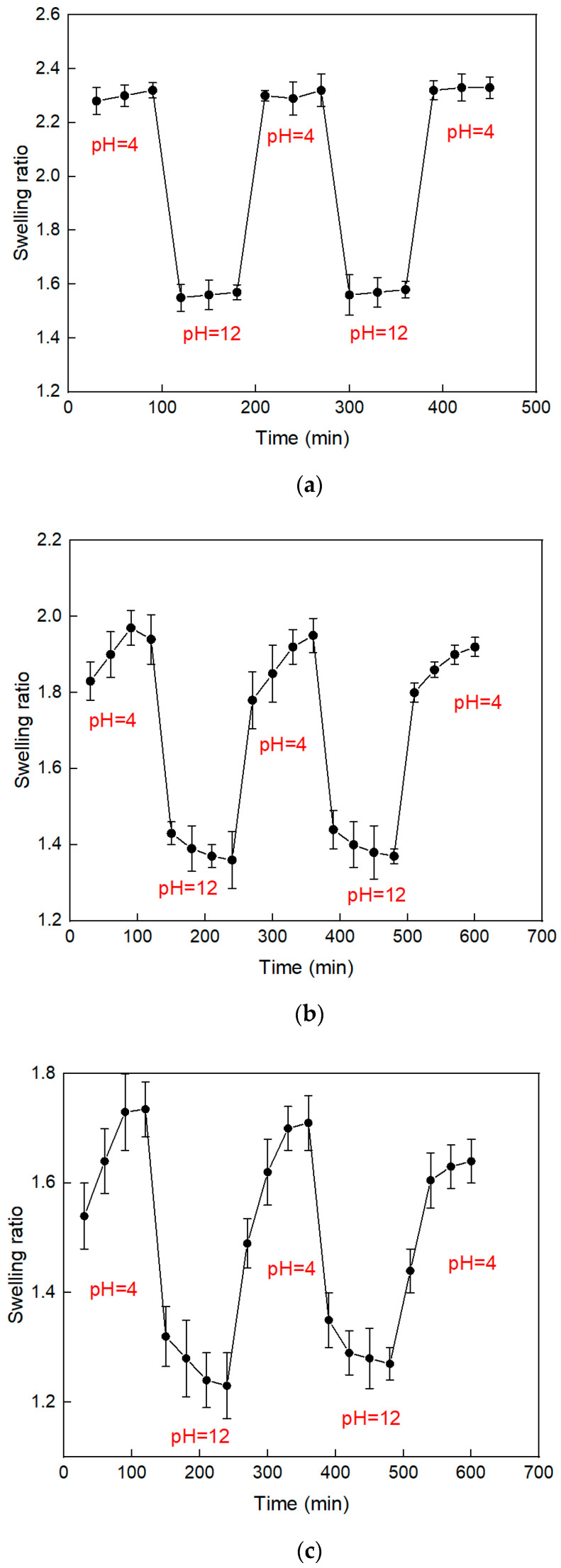
Swelling response of hydrogel (**a**) CG50, (**b**) CG75, and (**c**) CG90, in different pH solutions.

In order to confirm whether the Chitosan/GPTMS hydrogel can withstand a large degree of swelling and de-swelling behavior, in this experiment, the dried Chitosan/GPTMS hydrogel film was alternately placed in a hydrochloric acid/sodium hydroxide solution at pH = 4 and pH = 12. Among them, it was placed in an acidic and alkaline buffer solution for 90 min, and it was taken out and wiped to dry the surface every 30 min, and weighed, and repeated several times to test the pH response of the hydrogel. The experimental results found that CG10 and CG25 have lower crosslinking, making them less resistant to acids and alkalis, as well as the ability to withstand swelling and de-swelling. During the experiment, hydrogels could not withstand excessive environmental changes, leading to hydrogel film breaks, which in turn affect the experimental results. In other words, its mechanical properties are poor. According to Figure 6a, it can be found that CG50 hydrogel has a good reversible response to pH value, and the hydrogel has a good swelling ability in an acidic environment, because the external H^+^ ions enter in a low-pH environment. The inside of the hydrogel is combined with –NH to form –NH_2_^+^, which causes the repulsive force in the polymer network to increase, resulting in the molecular chain in an extended state, and the hydrogel has a noticeable swelling behavior due to the extension of the polymer chain.

When the hydrogel is placed in an alkaline environment, the deprotonation phenomenon will occur inside the colloid, which will cause the polymer chain to shrink and also make the hydrogel exhibit a de-swelling shape. It can also be seen from the experimental results that CG50 has sufficient mechanical properties to withstand large environmental changes. Figure 6b,c shows the response graphs of CG75 and CG90. It can be found that the response time is longer, mainly because the hydrogel porosity is lower, only allowing water molecules to enter the hydrogel at a slower rate. CG75 and CG90 hydrogels also have sufficient mechanical properties to withstand environmental changes.

#### 3.1.6. Mechanical Properties of Chitosan/GPTMS Hydrogel

Increasing the mechanical properties of Chitosan/GPTMS hydrogels is one of the main points of this study. Because Chitosan hydrogels have poor acid resistance and ability to protect themself against environmental changes, the addition of GPTMS increases the degree of crosslinking, in order to improve its mechanical properties.

**Figure 7:**
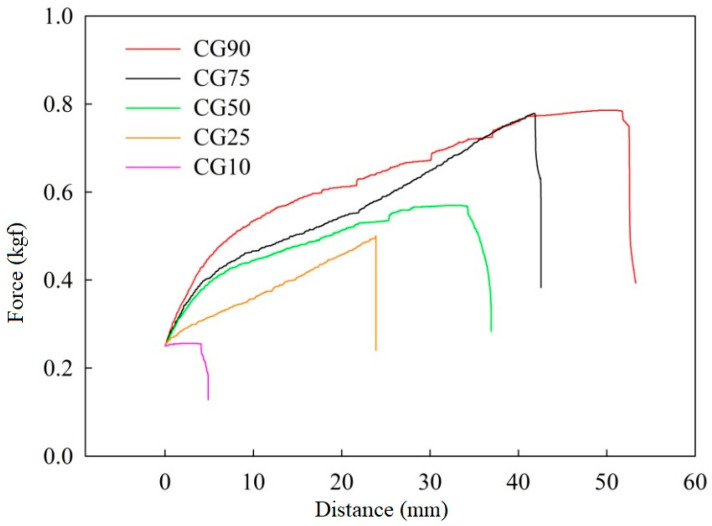
Tensile test chart of hydrogels with different proportions.

The experiment first measured the tensile area and thickness of the hydrogel, and then used a tensile strength tester to measure the mechanical strength of the hydrogel, at a test speed of 1 cm/min. The obtained elastic coefficient and tensile strength can be used to determine the mechanical strength of the hydrogel. The larger the value, the better the mechanical properties of the hydrogel. From Figure 7 and Table 4, it can be found that, as the content of GPTMS increases, the coefficient of elasticity and tensile strength of the hydrogel film also increase. It can be seen that, through the addition of GPTMS to improve the mechanical properties of Chitosan, the hydrogel is successful.

#### 3.1.7. Conclusion of Hydrogel Characteristics Analysis

Chitosan/GPTMS hydrogel films with pH sensitivity were synthesized, and their basic characteristics were analyzed. Based on the above results, the pH-sensitive hydrogel Chitosan/GPTMS hydrogel can be successfully polymerized through the crosslinking reaction and sol-gel method under acidic environment. The addition of GPTMS in Chitosan/GPTMS hydrogels results in an increase in the degree of crosslinking of the hydrogel and the increase in the number of hydrophobic groups, which increases the hydrophobicity and reduces the degree of swelling of the hydrogel. After the CG50 hydrogel passed the environmental response test, it was confirmed that it can have a good pH response in an acid and alkali solution, and it can withstand environmental changes without causing the hydrogel to break. It is a successful pH-sensitive hydrogel.

### 3.2. Thermodynamic and Kinetic Analysis of Chitosan/GPTMS Hydrogel

In this section, the melting endothermic peak of the heating curve of the DSC thermal analysis is substituted into the empirical formula, to calculate the overall content of water in the hydrogel after the water gel swells, and the tri-state water (free water, freezing bond water, and non-freezing bond water), and the relationship between the increase and decrease of water content and the structure of hydrogel was discussed.

Chitosan/GPTMS hydrogel will affect the degree of swelling of the hydrogel due to changes in the environmental pH value. The degree of swelling is closely related to the interaction between the polymer and the solvent. In this section, the swelling thermodynamics and swelling kinetics of Chitosan/GPTMS hydrogel films were studied. Through these two analyses, the relationship between the hydrogel polymer structure and swelling properties were explored. The thermodynamics part uses the Flory–Rehner theoretical equation to calculate the total interaction force parameter (χ) between the solvent and the polymer. The χ value refers to the evaluation parameter of the energy change caused by the interaction between the solvent and the polymer.

The swelling kinetics part is based on the empirical formula proposed by Peppas et al. to explain the hydrogel’s transport behavior during swelling [39]. Through obtaining the hydrogel’s kinetic parameters, such as swelling mechanism index (n) and the characteristic constant (k), the swelling behavior of the hydrogel during swelling, and the relationship between the diffusion behavior of the solvent into the hydrogel and the relaxation rate of the polymer chain can be explained.

#### 3.2.1. Analysis of Water Thermal Behavior State in Chitosan/GPTMS Hydrogels by DSC

The interaction between the hydrogel network and water has a great impact on its structure, function, and performance. Tri-state water also plays a very important role in the organism. The swelling of polymers in water can cause changes in physical properties (such as mechanics) and chemical properties. Therefore, the properties of hydrogels depend not only on the crosslinked structure of the polymer, but also on the presence of water in the interconnected structure and characteristics.

##### DSC Heating Curve

The polymer hydrogel can be divided into three stages in the swelling process. Part of the entering water molecules will first occupy the free space of the hydrogel, to form a free water form. The other part will bind to the hydrophilic group in the molecule, which is limited by the intramolecular hydrogen bonding; its form is non-freezing and cannot be detected by DSC. When the non-freezing water reaches a certain level or more, the combination of water molecules and hydrophilic groups in the molecule tends to be saturated, and the water molecules that enter again will enter the polymer network structure along the non-freezing water layer, forming a second layer or third layer of water-molecule clusters. These cage-like clusters enable water molecules to form the largest amount of hydrogen bonding with hydrogel molecules in the effective space in the hydrogel, so-called freezing bond water. Because the hydrogel molecular is swollen by the bond water to create more space, more water molecules can be allowed to continuously enter the hydrogel body, occupying it, that is, the free water content continues to increase and finally approaches saturation. The DSC heating curve was used to analyze the water molecule type in the hydrogel. The endothermic peak signal was the phase change of freezing bond and free water. Generally, the thawing temperature of water molecules is above 0 °C. Freezing bond water exhibits a lower thawing temperature (<0 °C) than normal free water. Non-freezing bond water does not present a phase change because it is a hydrogen bond formed with a hydrophilic group in a hydrogel molecule.

Figure 4 shows the DSC heating curve of Chitosan/GPTMS hydrogels with different formulations, starting from −25 °C. When a small amount of GPTMS is added, the phenomenon of coexistence of freezing bond water (<0 °C endothermic peak) and free water can be seen in the endothermic peak near 0 °C, and the addition of more GPTMS will cause the intramolecular hydrogen bonding effect to be enhanced, and the freezing bond water can be converted into free water. Therefore, the endothermic peak in the figure will exhibit the characteristics of free water. On the other hand, the moisture content of the hydrogel also tends to decrease with the increase of the content of GPTMS. This is because the addition of GPTMS causes the hydrophobic group of the hydrogel to increase (Si–O–Si) group, as well as increasing the intramolecular hydrogen bonding effects.

##### Growth and Decline of Freezing Water (W_fm_) and Non-Freezing Water (W_nf_) Contents in Hydrogels

Since the state of water in the gel is related to the interaction of hydrogel/water molecules, the effect of the composition of the hydrogel on water cannot be ignored. The state and content of water in the Chitosan/GPTMS hydrogel are shown in Table 5. It is shown that the content of “three-state water” in hydrogels will increase or decrease due to the content of GPTMS. Generally speaking, in the non-freezing state, water molecules and the –NH and –OH functional groups on the Chitosan/GPTMS hydrogel molecular chain simultaneously produce the tightest hydrogen bonding. It is known from Table 5 that the non-freezing bond water of each component of the hydrogel is CG10 (43.43%), CG25 (37.81%), CG50 (36.82%), CG75 (35.88%), and CG90 (33.74%). The reason is that, when the GPTMS is added too much, the crosslinking density of the hydrogel increases, resulting in a decrease in the mesh size and porosity of the polymer. The increase of GPTMS content leads to the increase of hydrogel hydrophobic groups (SiO_2_), and also reduces the bulk water content (BWC) and the freezing bond water content (W_fm_). Based on the analysis of the swelling degree and DSC thermal analysis, it can be found that Chitosan/GPTMS hydrogel does indeed cause the rise and fall of water molecules’ states in the hydrogel due to the interaction between the polymer segments in the structure and the water molecules.

#### 3.2.2. Thermodynamic Analysis of Swelling of Chitosan/GPTMS Hydrogel

##### Analysis of Swelling Structure Parameters of Hydrogel

The molecular weight between the crosslinking points in the hydrogel mesh can also be used to indicate the effective crosslinking density of the hydrogel. It is known from the calculation results of Equations (5)–(8) (Table 6) that, when the content of GPTMS in the hydrogel increases, the molecular weight between the crosslinking points decreases, representing an increase in the effective crosslinking density; while the distance between the crosslinking points of the hydrogel becomes smaller, the mesh size of the gel is also reduced, so it can be observed that CG90 has the smallest mesh size in any environment. When the hydrogel is placed in an acidic environment, due to the hydrogel –NH group internally accepts protons to form –NH_2_^+^, which increases the electrostatic repulsion and causes the hydrogel molecular chain to expand. Therefore, it is also observed that the mesh size is greater than that of DI water and alkaline environment.

As shown in Table 6, the polymer volume fraction during swelling will increase with the increase of GPTMS content in the hydrogel. The main reason is that the addition of GPTMS reduces the hydrophilicity of the hydrogel, which reduces the amount of water that can be contained in the polymer network. Looking at the porosity results of the hydrogel (Table 6), it can be observed that increasing the content of GPTMS and placing the hydrogel in an alkaline solution results in a decrease in the porosity of the hydrogel, so that water molecules slowly penetrate into the hydrogel, and it also represents the slower the solvent diffusion rate.

##### Influencing Factors of the Interaction Force Parameter (χ)

Interaction force parameter (χ) value can be seen as an indicator of the interaction between the polymer gel and the solvent, which can be roughly regarded as the hydrophilic and hydrophobic ability of the polymer in the solvent.

The force parameter is derived from Flory–Huggins theory [36]. After subsequent research and calculation, the force parameter expression of Equation (6) can be obtained.

##### Effect of Hydrogel Composition on Interaction Force Parameters

From the results in Table 7, it can be found that the value of χ increases with the increase of the GPTMS content, and the value of χ increases from 0.6051 to 0.627 with the increase of the GPTMS content if hydrogel in DI water. The main reason is that, after adding more GPTMS, the hydrophobic groups increase, which also increases the intramolecular hydrogen bonding effect, and the degree of crosslinking also increases with the increase of GPTMS, which is against the molecular chain expansion, so the χ value will increase. The value of χ can be roughly regarded as the hydrophilic and hydrophobic ability of the polymer in the solvent. A higher value of χ indicates that the interaction between the polymer and water is weak, and the interaction between the hydrophobic groups or the polymer chains is stronger. In other words, CG10 has better hydrophilicity in the solvent, while CG90 has relatively poorer hydrophilicity. This result also agrees with the degree of swelling of the hydrogel. When the hydrogel is placed in a solution with a different pH value, it can also be found that the χ value is smaller in an acidic environment, indicating that the hydrogel and the solvent have a stronger interaction force.

#### 3.2.3. Swelling Kinetics of Chitosan/GPTMS Hydrogel

In the process of hydrogel swelling, the rearrangement of polymer segments often affects the absorption or penetration of the hydrogel. According to the literature [37,38], the explanation of hydrogel swelling kinetics can be roughly divided into over-swelling phenomenon and Fick ’s diffusion. The two interpretation angles are different, but the essence is not in conflict. One is to explain the phenomenon of over-swelling from the structural factors, including the external shape of the hydrogel, and the other is to control the diffusion mechanism through the relaxation of the polymer chain, to explain the hydrogel’s swelling process. Both of the above swelling kinetics mechanisms can be attributed to the synergistic physical crosslinks generated by the interaction between functional groups during hydrogel swelling, such as hydrogen bonding, hydrophobic interactions, ionic bonding, Van der Waals forces, and charge transfer.

##### Dynamic Swelling Curve of Hydrogel

**Figure 8:**
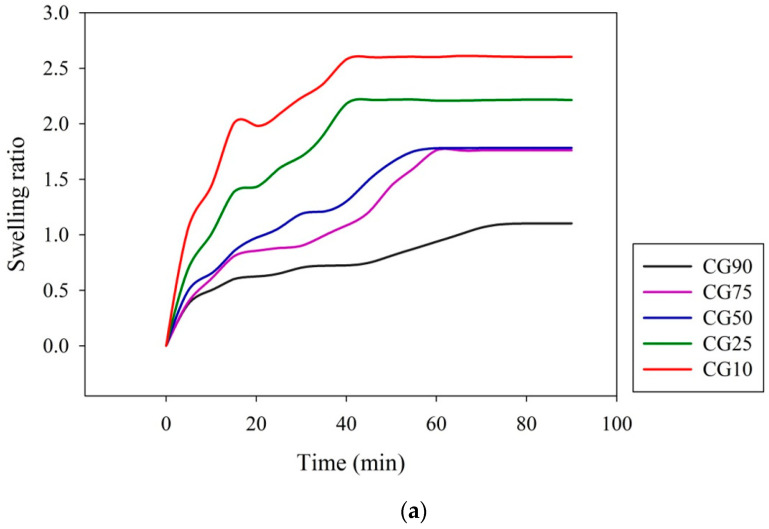

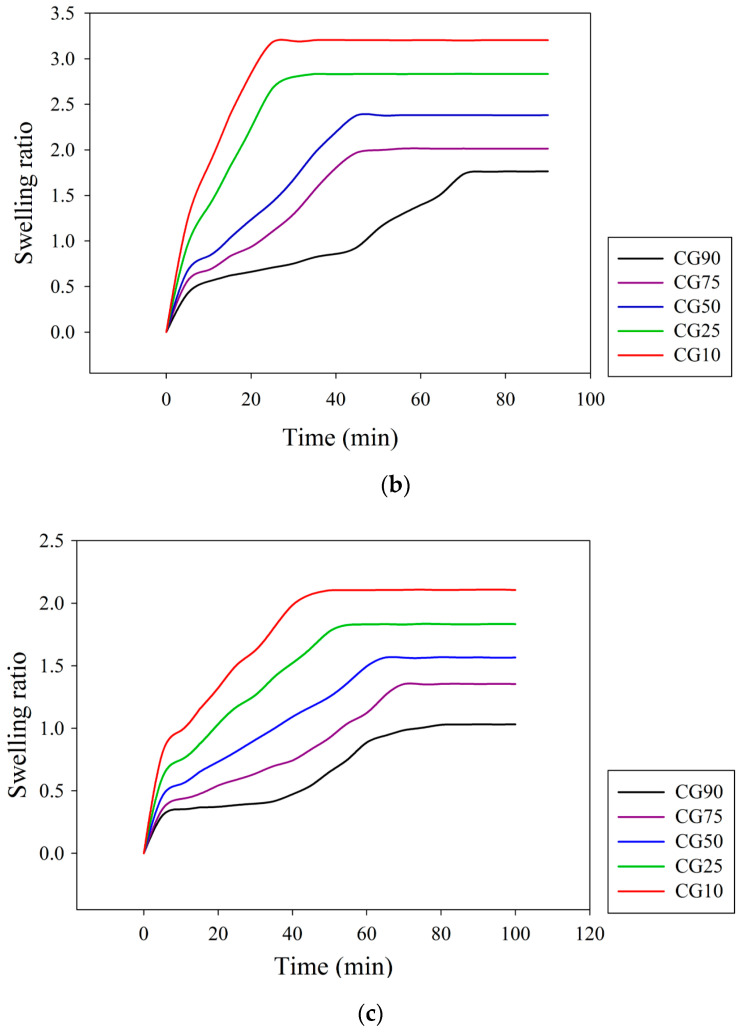
Effect of different ratios of Chitosan/GPTMS hydrogels on (**a**) DI water; (**b**) pH = 4; (**c**) pH = 12 on dynamic equilibrium swelling.

Figure 8 shows the dynamic swelling curves of Chitosan/GPTMS hydrogels with different GPTMS ratios under different pH environments. It can be found from the figure that the time required for CG10 to reach the maximum swelling degree is shorter, and as more GPTMS is added, the time required to reach equilibrium swelling is longer. The reason may be that, as the degree of crosslinking of the hydrogel increases, the rate at which water molecules enter the hydrogel is also affected and becomes slower. The degree of equilibrium swelling will also be affected by the intramolecular hydrogen bonding effect, the effect of hydrophobic groups, and the degree of crosslinking. When the hydrogel is placed in an acidic environment, because external H^+^ ions enter the hydrogel and combine with –NH to form –NH_2_^+^, the repulsive force in the polymer network increases, resulting in the molecular chain being stretched, and makes the hydrogel exhibit obvious swelling behavior and a faster swelling rate. When the hydrogel is placed in an alkaline environment, the deprotonation phenomenon will occur inside the colloid, causing the polymer chain to shrink and make the hydrogel de-swelling state.

##### Swelling Mechanism of Hydrogel

By plotting the dynamic equilibrium expansion rate against time, it can be determined that the swelling process of the Chitosan/GPTMS hydrogel does not experience over–swelling effects. According to the literature [33], the Fick’s diffusion equation can be used for the dynamic swelling mechanism of thin–film hydrogels to describe the initial swelling behavior, as in Equation (7).

The dynamic swelling mechanism (n) of hydrogels can be defined by Fick’s diffusion equation at 60% below the maximum equilibrium swelling rate. The value of n can be used to explain the correlation between the relaxation rate of the hydrogel polymer chain and the solvent diffusion rate. When the value of *n* is less than or equal to 0.5, Fick’s diffusion dominates the swelling process, which is the most ideal mechanism. When the value of *n* is between 0.5 and 1, it is non-Fick’s diffusion.

From Table 8, it can be observed that, with the exception of CG10 and CG25, the dynamic swelling mechanism index *n* values of other proportions of hydrogel films are less than 0.5, indicating that the main mode of CG50, CG75, and CG90 are solvent diffusion during the initial swelling process of hydrogel. The *n* values of CG50 placed in acidic and DI water are 0.46 and 0.48, which are closer to the ideal Fick’s diffusion. This also means that the permeation rate of water can be controlled. All hydrogel samples have smaller values of *n* when they are placed in an alkaline environment. This indicates that the mobility of the hydrogel molecular chain is reduced, and the affinity between the water molecule and the hydrogel film is small, causing the water molecules to diffuse into the hydrogel at a slower rate.

Chitosan/GPTMS hydrogel has better swelling ability and higher tri-state water content when adding a small amount of GPTMS. It is known through thermodynamic calculations that the force parameters are also in line with the DSC heating curve. After adding more GPTMS, the interaction force between the hydrogel and the solvent is decreased, that is, the hydrophilic force between the solvent and the hydrogel is decreased. Kinetic analysis of the hydrogel’s swelling mechanism demonstrates that, when the hydrogels were placed in an alkaline environment, the value of *n* was very different from the best swelling mechanism, indicating that the mobility of the hydrogel molecular chain was low. The affinity between water molecules and hydrogel films is small; thus, it is not conducive to the diffusion of water. In acidic and DI water, the *n* value of CG50 film is closer to 0.5, which indicates that it is most in line with Fick’s diffusion mechanism, and it also means that the water permeation rate can be controlled.

## 4. Conclusions

Chitosan and GPTMS were subjected to ring-opening and sol-gel reactions in an acidic environment, to successfully synthesize biodegradable pH-sensitive Chitosan/GPTMS hydrogel films. A total of 50 wt.% of GPTMS was added into the chitosan hydrogel, to form CG50 hydrogel, which was then placed in the solution of pH = 4 and pH = 12, alternately, and had good pH response ability and sufficient mechanical properties to cope with the rapid changes of the environment. The amount of freezing and non-freezing bond water in the hydrogel was obtained by DSC thermal analysis. The increase of GPTMS content led to an increase in the degree of crosslinking of hydrogels and an increase in hydrophobic groups, thus reducing the freezing and non-freezing bond water. The degree of swelling of Chitosan/GPTMS hydrogels was decreased due to the increase of GPTMS content, and the value of the interaction force parameter (χ) was also increased with the increase of GPTMS content, indicating that the repulsive force between the hydrogel and the solvent was increased. When the hydrogel is placed in an acidic environment, the value of χ is low, which means that the hydrogel is protonated by the solvent and has strong mutual attraction. The gel swelling structure parameters can be known in a fixed pH solution; the increase of GPTMS in the hydrogel led to a decrease in the molecular weight between the crosslinking points and a smaller distance between the crosslinking points of the hydrogel, so the hydrogel mesh size and porosity were also reduced. After the CG50 hydrogel is tested by the swelling kinetics, it can be found that its swelling mechanism index approaches 0.5, which agrees with the Fickers diffusion mechanism, and it also means that the water penetration rate can be regulated. The hydrogels (CG50) used in this study have good environmental sensitivity and mechanical properties and are suitable for the use of drug release or biomedical applications.

## Figures and Tables

**Table 1:** Equilibrium swelling degree of Chitosan/GPTMS hydrogels with different ratios.

	CG10	CG25	CG50	CG75	CG90
Swelling ratio	2.60 ± 0.11	2.22 ± 0.13	1.78 ± 0.1	1.76 ± 0.12	1.59 ± 0.09

**Table 2:** Cracking temperature and inorganic portion residual weight of different GPTMS ratio of hydrogels.

	Chitosan	GPTMS	CG10	CG25	CG50	CG75	CG90
T_d_ (°C)	250	303	259	260	261	269	272
Residual weight (wt.%)	28.13	49.57	36.18	37.40	38.47	40.30	42.20

**Table 3:** Equilibrium swelling degree of Chitosan/GPTMS hydrogel in different pH solutions.

	Swelling Ratio				
	CG10	CG25	CG50	CG75	CG90
pH = 4	3.204 ± 0.24	2.833 ± 0.11	2.381 ± 0.08	2.014 ± 0.1	1.765 ± 0.11
pH = 12	2.108 ± 0.13	1.832 ± 0.16	1.567 ± 0.11	1.354 ± 0.21	1.221 ± 0.14

**Table 4:** Tensile strength and elastic coefficient of each proportion of hydrogel.

Sample Name	Coefficient of Elasticity (N/cm^2^)	Tensile Strength (kgf/cm^2^)
CG10	1.297	2.563
CG25	2.555	5.007
CG50	4.119	5.703
CG75	4.571	7.788
CG90	4.861	7.864

**Table 5:** State and content of water in Chitosan/GPTMS hydrogel.

	CG10	CG25	CG50	CG75	CG90
BWC (%)	81.93	69.85	64.08	61.88	51.81
W_fm_ (%)	38.50	32.04	27.25	26.00	18.07
W_nf_ (%)	43.43	37.81	36.82	35.88	33.74

**Table 6:** Swelling structure parameters of hydrogel.

pH	Sample	$\phi_{2}$	M_c_ (g/mol)	ξ (Å)	ε
4	CG10	0.231	13267.94	155.52	1.59
	CG25	0.252	11747.64	128.38	1.56
	CG50	0.264	9823.57	115.59	1.52
	CG75	0.276	8256.91	104.41	1.48
	CG90	0.294	6422.12	90.168	1.44
6.7(Di water)	CG10	0.315	4854.25	76.61	1.34
	CG25	0.336	3717.86	65.62	1.14
	CG50	0.363	2685.02	54.35	1.04
	CG75	0.366	2592.55	53.26	1.01
	CG90	0.381	2182.62	48.21	0.84
12	CG10	0.606	1253.37	14.07	0.81
	CG25	0.612	1170.93	13.67	0.77
	CG50	0.621	1123.50	13.11	0.73
	CG75	0.630	1107.43	12.56	0.69
	CG90	0.642	1087.89	11.88	0.66

**Table 7:** Parameters of interaction force between hydrogel and solvent.

			χ		
pH	CG10	CG25	CG50	CG75	CG90
**4**	0.577	0.584	0.588	0.592	0.598
**6.7 (DI water)**	0.605	0.612	0.621	0.622	0.627
**12**	0.702	0.704	0.707	0.710	0.714

**Table 8:** Effects of different formulations of hydrogels on swelling mechanism (n) under different environments.

pH	Sample	n	K (×10^−2^)	R^2^
**4**	CG10	0.60	43.40	0.998
	CG25	0.58	41.42	0.996
	CG50	0.46	40.81	0.974
	CG75	0.41	42.43	0.971
	CG90	0.34	42.62	0.987
**6.7** **(Di-water)**	CG10	0.55	42.88	0.985
	CG25	0.54	41.69	0.983
	CG50	0.48	40.73	0.992
	CG75	0.44	39.91	0.983
	CG90	0.30	41.82	0.980
**12**	CG10	0.35	45.92	0.987
	CG25	0.37	45.24	0.986
	CG50	0.38	44.25	0.982
	CG75	0.33	44.11	0.988
	CG90	0.21	43.41	0.983
